# Letter to the editor. Gene editing and disabled people: a response to Iñigo de Miguel Beriain

**DOI:** 10.1007/s12687-020-00465-5

**Published:** 2020-04-30

**Authors:** Felicity Boardman

**Affiliations:** Warwick Medical School, Gibbet Hill Road, Coventry, CV4 7AL UK

The response of disabled people to the possibility of human germline genome editing (HGGE) is an important topic, and one recently presented in an article by Kleiderman and Kellner Stedman (2020). The need to engage directly with members of the disability and rare disease community on such developments in genomic medicine was further underscored by my recent editorial (Boardman 2020), within which I highlighted the role of identity politics in informing reactions to HGGE amongst such groups who live directly with genetic conditions.

Writing in reaction to this editorial, Iñigo De Miguel Beriain (2020) has defended the use of HGGE against some of the critiques levelled by disability rights supporters that I outlined, by appealing to the principle of human dignity. He does this through the use of two key points:

1. That life without a disability should be regarded as preferable to one with a disability.
2. That if we were to accept that it is in the best interests of wider society to retain disability as a trait in its members, that ‘forcing people to suffer’ (De Miguel Beriain 2020) with disabilities to meet this end is unethical, because this would mean that the ‘welfare of human beings [would] be sacrificed for the good of the collective’, which, he argues, is not in keeping with the principle of human dignity.

I would like to respond to De Miguel Beriain’s arguments by firstly highlighting the erroneous assumptions inherent in his first point, before showing how the second no longer stands once the first is refuted.

The argument that life *without* disability must surely be preferable to one *with* disability is not new, and one certainly that is largely upheld within the beliefs and reproductive practices of wider society. However, I dispute the claim, made directly by De Miguel Beriain, that the inferiority of life with disability to one without it is ‘an acceptable conclusion for everyone’ (De Miguel Beriain 2020).

There are numerous examples of people with genetic conditions who not only readily accept a child with the same condition as themselves (Henley 2016; Black 2016; Lancaster 2011) but also sometimes actively seek it (Teather 2002; Shanghavi 2006). A study by Baruch and colleagues, for example, found that 3% of the 137 IVF centres they surveyed in the USA had used PGD to actively select *for* the disabling trait of the parents (Baruch et al. 2008). For some would-be parents, there are practical considerations (e.g. linguistic, environmental) that inform a decision to select *for* disability, or, at least, refrain from preventing it (Shanghavi 2006). Yet for others, the decision stems from a fundamental conviction that life with a disabling condition is intrinsically of no less value than a life without it (Wallis 2019). As such, the physical, financial and psychological risks associated with technologically mediated reproduction are not justified.

In his response, De Miguel Beriain (2020) does acknowledge, however, that there are some disabled people who are reluctant to accept treatments for their condition, and as such may not welcome HGGE technologies. This group of people he refers to as ‘type A patients’, in contrast to ‘type B patients’, a category he reserves for those disabled people who would prefer removal of their condition. Sticking with this taxonomy, I accept De Miguel Beriain’s statement that it is difficult—if not impossible–to prenatally predict which response (type A or type B) a would-be-disabled embryo will adopt to their condition. Experience of genetic disability is always mediated by social, cultural and environmental factors, healthcare availability and access, and psychological differences between individuals—factors that cannot be pre-determined by a genetic test.

Despite this complexity, however, research is increasingly demonstrating that there are a range of factors that make a ‘type A’ response to disablement more likely. Amongst which, early/congenital onset is a significant factor (Bogart 2014, 2019; Hahn and Belt 2004; Shakespeare 2006; Boardman et al. 2017; Jamoom et al. 2008). People who are born with their disability are more likely to have incorporated their condition into their identity and sense of self, and to have set their lives up around its existence than those who firstly experience able-bodiedness before becoming disabled. People with later onset disabilities typically undergo significant (and sometimes recurrent) identity negotiations and life re-structuring as they adapt to their disability, which may involve varying degrees of loss (Shakespeare 2006). It is these people who are much more likely to externalise their disability and separate it from their sense of self (Watson 2002). Such individuals are therefore more likely to view their condition as an unwelcome intrusion in their lives and to desire its eradication. These individuals are therefore far more likely to fall into the ‘type B’ category that De Miguel Beriain (2020) had in mind.

De Miguel Beriain (2020) has argued that it is these people—with experience of both being able-bodied *and* disabled—who can give us the greatest insight into the best interests of the would-be-disabled embryo in HGGE contexts. However, I suggest that we instead need to focus our attention on the experiences and perspectives of those whose conditions are early onset, many of whom (though clearly not all) will be ‘type A patients’. As De Miguel Beriain himself points out, the single gene disorders that will most likely be targeted by HGGE are typically early onset in presentation; therefore, it is *these* people’s lives who most realistically mirror those to be expected for the would-be-disabled embryos.

By highlighting this emerging body of research that demonstrates that there are factors that make a type A or type B response to genetic disability more likely, I am not suggesting that responses to disability can be predicted. Rather, we need to acknowledge the complexity of lived experience with genetic disability and the importance of not essentialising that experience as universally negative and always to be avoided. Indeed, as social model of disability theorists has long pointed out, there are a range of factors that have a significant impact on the experience of disablement that are entirely unrelated to the impairment itself (Barnes and Mercer 2004; Boardman et al. 2020).

We should be careful not to assume that disabled people who do not wish to use reprogenetic technologies in order to avoid disability, or who otherwise value their lives positively (perhaps by refusing therapies), are somehow misguided, or unrepresentative of the views of the wider population of disabled people. Indeed, research continues to highlight what has come to be termed the ‘disability paradox’, that is, that disabled people consistently rate their quality of life higher than others do around them (Albrecht and Devlieger 1999). At a time when disabled people are afforded greater support, access to healthcare and legal protections than ever before in history, the calculation of whether life with disability is inherently worse than one without cannot be treated as a one-off decision as it is so closely bound to the social and environmental context in which that life will be lived out.

De Miguel Beriain’s second argument, that we should not inflict suffering (through disability) onto people for the greater good of society, is entirely defeated when we unshackle disability from its association with suffering. This is not to deny that genetic conditions can cause suffering—they can, and they invariably do. Moreover, as we have established, there is no sufficiently reliable way to distinguish between those who will suffer with their condition and those who will thrive. However, whilst De Miguel Beriain argues that, given this uncertainty, we should err on the side of editing genomes, we also need to take seriously the harms that are inflicted when we perpetuate and reinforce a presumed causal relationship between disability and suffering. This pairing not only impedes a thorough consideration of the range of human experiences that HGGE will impact, but also harms currently existing disabled people, in a variety of ways.

When De Miguel Beriain states that a person, or society, is unlikely to ‘miss out on anything’ by no longer having SMA or haemophilia, I would point to the testimonies of people who live with these conditions, and whose personalities and values (and those of the people around them) have been shaped, in various ways, by experiencing them (Boardman et al. 2017; Boardman and Hale 2018; Boardman et al. 2019)—even in cases where the condition was described as involving significant suffering (see ‘Annette’ (pp. 190–191) in Boardman 2017). Indeed, terms like ‘Deaf gain’ have emerged to describe the ‘…unique cognitive, creative, and cultural gains’ that are brought about through Deaf ways of being in the world (Bauman and Murray 2014) and draw attention to the possibility of disability being an enriching experience, both at the individual and societal level.

It is my view that we firstly need to better understand what we mean by the term ‘suffering’ before we can presume to apply it to lives not yet lived. The accounts of genetically disabled people can help us delineate where the boundaries of suffering might lie, in order to better regulate the appropriate uses of technologies such as HGGE. Indeed, it is thoroughly in keeping with, and even integral to, the principle of human dignity to respect and uphold the value that people assign to their own lives—even if they contradict our own.
